# Behavioral Adaptation and Response to Pulmonary Rehabilitation in Older Patients with Advanced COPD: Beyond Clinical and Immune Biomarkers

**DOI:** 10.3390/arm94050074

**Published:** 2026-10-19

**Authors:** Patrizia Russo, Stefania Proietti, Laura Vitiello, Simona Pace, Carlo Tomino, Giorgio Felzani, Giulia Prinzi, Stefano Bonassi

**Affiliations:** 1 Department of Human Sciences and Promotion of the Quality of Life, San Raffaele University, Via di Val Cannuta 247, 00166 Rome, Italy; patrizia.russo@uniroma5.it (P.R.); simona.pace@uniroma5.it (S.P.); stefano.bonassi@sanraffaele.it (S.B.); 2 San Raffaele Sulmona, Viale dell’Agricoltura, 67039 Sulmona, Italy; carlo.tomino@sanraffaele.it (C.T.); giorgio.felzani@sanraffaele.it (G.F.); 3 AgEA Coordinating Body, Via Palestro 81, 00185 Rome, Italy; s.proietti@agea.gov.it; 4 Clinical and Molecular Epidemiology, IRCCS San Raffaele Roma, Via di Val Cannuta 247, 00166 Rome, Italy; 5 Mental Health Department, ASL RM3, Via Giancarlo Passeroni, 28, 00122 Rome, Italy; giulia.prinzi@gmail.com

**Keywords:** chronic obstructive pulmonary disease, pulmonary rehabilitation, behavioral adaptation, coping strategies, Brief COPE, rehabilitation response, psychological support, aging

## Abstract

**Highlights:**

**What are the main findings?**

- Planning and active coping were associated with greater functional improvement after pulmonary rehabilitation in older patients with advanced COPD.
- Planning was the only coping strategy showing exploratory evidence compatible with mediation of the association between age and rehabilitation response.

**What are the implications of the main findings?**

- Assessing coping strategies before pulmonary rehabilitation may complement clinical and functional evaluation and help identify patients who could benefit from tailored psychological support.
- The potential value of coping-based interventions in improving and maintaining rehabilitation outcomes should be investigated in larger prospective studies.

**Abstract:**

Patients with advanced chronic obstructive pulmonary disease (COPD) respond differently to pulmonary rehabilitation (PR), and respiratory impairment alone might not fully explain this variability. Behavioral adaptation may contribute to differences in rehabilitation response. We studied 102 patients with advanced COPD (GOLD III–IV) who completed a 3-week multidisciplinary PR program. Functional response was assessed by change in six-minute walking distance (6MWD). Clinical, functional, psychological, lifestyle, and immune characteristics were evaluated, including coping strategies measured with the Brief COPE, Torque Teno virus (TTV) viremia, and circulating T-cell subsets. Exploratory mediation analyses examined whether coping strategies contributed to the association between age and rehabilitation response. Greater improvement in 6MWD was associated with higher active coping and planning scores, while self-distraction also differed across response groups. Planning scores were higher in good responders than in non-responders (7.10 ± 1.35 vs. 5.05 ± 1.93, respectively; *p* < 0.001). Planning was the only coping strategy showing a statistically significant indirect effect in the exploratory mediation analysis. The estimated proportion mediated was approximately 26.5%, although the wide confidence interval indicated substantial uncertainty. These findings should be interpreted cautiously given the observational design and the assessment of multiple coping strategies. Conventional physiological measures, lifestyle characteristics, and immune biomarkers—including TTV viremia and circulating T-cell subsets—did not significantly distinguish rehabilitation response groups. Planning and active coping were associated with response in both sexes. A separate pilot randomized study, IMPROVE (*n* = 36), was subsequently conducted to investigate whether structured psychological support could influence coping strategies and help maintain the benefits of PR. At six months, patients receiving psychological support showed better maintenance of rehabilitation benefits, alongside improvements in psychological well-being, quality of life, and selected coping strategies. These findings provide preliminary evidence that coping-related characteristics may be modifiable; however, IMPROVE was a separate exploratory intervention study and was not designed to validate the observational findings. Assessment of coping strategies may complement clinical and functional evaluation and help identify patients who could benefit from tailored psychological support. Their clinical utility and the effectiveness of targeted interventions require confirmation in larger prospective studies.

## 1. Introduction

Chronic obstructive pulmonary disease (COPD) is a heterogeneous systemic disorder characterized by persistent airflow limitation and substantial variability in clinical presentation, disease progression, and treatment response [1]. Its clinical expression reflects the interaction of respiratory impairment with comorbidities, environmental exposures, and individual biological and behavioral characteristics [2,3]. This heterogeneity is particularly evident in patients with similar degrees of airflow limitation but different levels of disability, quality of life, and response to treatment.

Pulmonary rehabilitation (PR) is a cornerstone of non-pharmacological treatment for symptomatic COPD. Exercise training, education, and nutritional and behavioral support improve exercise capacity, health-related quality of life, psychological well-being, and self-management [4,5,6,7,8,9,10]. However, the response to PR varies considerably among patients, and lung function alone does not adequately explain these differences. Functional status, comorbidities, psychological distress, and other patient characteristics may all contribute to rehabilitation outcomes [7,11,12].

Psychological factors are particularly relevant. Anxiety and depression are common in COPD and are associated with poorer functional status and quality of life [13]. Yet emotional distress does not fully capture how patients respond to the demands of chronic disease. Coping strategies may be more directly related to how patients engage with rehabilitation, manage symptoms, and maintain behavioral changes over time. Adaptive strategies such as active coping, planning, acceptance, and appropriate use of social support have been associated with better self-efficacy, treatment adherence, and participation in PR, whereas avoidant strategies tend to be associated with less favorable outcomes [14,15,16,17,18]. Coping may also differ between women and men. Women have more often been described as using emotion-focused strategies, whereas men tend to rely more on problem-focused and instrumental coping [19,20,21]. These differences may be relevant in COPD, where sex is also associated with differences in symptoms, comorbidities, and disease phenotype [3].

Coping is related to behavioral resilience, understood as the ability to maintain effective psychological and behavioral functioning despite persistent physiological and emotional stress [22,23]. However, coping strategies and resilience are not interchangeable concepts, and the extent to which specific coping processes contribute to the response to PR remains insufficiently understood.

There is also a biological basis for individual differences in stress adaptation. Interactions among genetic, epigenetic, and environmental factors contribute to the regulation of adaptive responses [24]. Among the genes involved in stress regulation, FKBP5 has attracted particular interest because of its role in glucocorticoid receptor signaling. FKBP5 variants have been associated with resilience, cognitive function, anxiety, and depression [25]. In elderly patients with COPD undergoing PR, the FKBP5 rs4713916 GA genotype was previously associated with more adaptive coping, better psychological well-being and quality of life, and greater rehabilitation responsiveness than the GG genotype [25]. These findings support a possible link between stress regulation, behavioral adaptation, and functional recovery.

Behavioral characteristics should, however, be considered alongside the biological burden of COPD. Chronic inflammation, oxidative stress, immune dysregulation, and biological aging contribute to disease progression and functional decline [2]. In the same clinical research program, higher Torque Teno virus (TTV) viremia, a potential marker of impaired immune competence, was associated with genomic damage and poorer long-term survival [26], while dietary habits were also related to rehabilitation responsiveness [27]. These observations suggest that rehabilitation outcomes may reflect the combined influence of functional, biological, and behavioral factors [28].

This multidimensional perspective is also supported by Candia et al. (2025) [29], who reviewed the potential role of biomarkers in exercise-based pulmonary rehabilitation for COPD. Their review highlighted the possible contribution of systemic inflammation, oxidative stress, and markers of DNA damage to the assessment of rehabilitation outcomes. The authors also discussed emerging candidates, including metabolic and microbiome-related markers, while emphasizing that findings remain inconsistent across studies and that further standardized, longitudinal research is needed. Importantly, the variability of biomarker responses reinforces the difficulty of explaining rehabilitation outcomes through a single biological measure. Although Candia et al. focused primarily on biological markers rather than coping strategies, their review supports a broader approach in which clinical, functional, and biological information is considered together when investigating individual differences in rehabilitation response.

Against this background, the present study investigated whether behavioral adaptation, assessed through specific coping strategies, was associated with pulmonary rehabilitation responsiveness in elderly patients with advanced COPD—beyond conventional clinical and functional measures. We examined the relationship between coping strategies and functional outcomes and explored whether planning might help explain the association between age and rehabilitation response. In a separate, subsequent pilot study, IMPROVE, we also investigated whether structured psychological support could modify coping-related characteristics and help maintain the benefits of rehabilitation over time. We hypothesized that patients reporting greater use of adaptive coping strategies would show better functional improvement and that structured psychological support might help sustain rehabilitation benefits. The IMPROVE study was designed as complementary exploratory interventional research, not as a validation cohort for the primary observational findings.

## 2. Materials and Methods

### 2.1. Study Design

The present study was based on an observational cohort of patients with advanced COPD referred to IRCCS San Raffaele, Rome, for inpatient pulmonary rehabilitation. The primary observational cohort included 102 consecutive patients recruited between 2013 and 2015. The observational study focused on the association between behavioral adaptation, clinical and biological characteristics, and response to pulmonary rehabilitation. At a later stage of the research program, the IMPROVE pilot study was conducted in a separate group of patients to investigate the potential contribution of structured psychological support to the maintenance of rehabilitation benefits and to changes in coping strategies. IMPROVE (patients recruited in 2017) was therefore designed as a complementary interventional pilot study and was not part of the original observational cohort or intended as a validation cohort. The two study components consequently differed in their recruitment period, population, and study objectives.

### 2.2. Primary Study

The primary study included 102 consecutive elderly patients with stable advanced COPD (GOLD III–IV) admitted to IRCCS San Raffaele, Rome. Patient characteristics and recruitment procedures have been described previously [25,26,27,28]. Eligible patients had stable COPD and were able to communicate in Italian and complete the study assessments. Exclusion criteria included active cancer, MMSE < 21, acute exacerbation, unstable cardiovascular disease, severe neurological disorders, or inability to participate in or complete the rehabilitation program.

Patients were referred to pulmonary rehabilitation after hospitalization for a COPD-related clinical event and after clinical stabilization. COPD diagnosis and disease severity had been established during the preceding hospital and specialist evaluation and were documented in the clinical records before admission to rehabilitation. Pulmonary function assessment, including spirometry, was part of the preceding clinical evaluation. Thus, admission to the rehabilitation program represented a subsequent phase of the clinical care pathway rather than the initial diagnostic assessment of COPD.

The study was conducted between 2013 and 2015 within the respiratory rehabilitation pathway of the Lazio Regional Health Service. During the study period, the applicable regional framework was the *Decreto del Commissario ad Acta* n. U00008 of 10 February 2011, which modified the requirements established by DCA U0090/2010 [30]. Annex C addressed respiratory rehabilitation and included access to pulmonary functional diagnostics, including basic spirometry, measurement of VEMS/FEV1, the Tiffeneau index, and oximetry [31].

The study was conducted in accordance with the Declaration of Helsinki and was approved by the Institutional Ethics Committee (Protocol 15/2013). All participants provided written informed consent. Vital status and causes of death were obtained from municipal registries during a 5-year follow-up. Blood and urine samples were collected at admission and stored at −80 °C until analysis.

#### 2.2.1. Pulmonary Rehabilitation Program

Patients underwent a standardized 3-week multidisciplinary inpatient pulmonary rehabilitation (PR) program according to the organizational framework of the Lazio Regional Health Service in force during the study period. The multidisciplinary team included pulmonologists, physiotherapists, psychologists, nurses, and nutritionists. The programme included individualized and progressively increased aerobic and resistance exercise training combined with structured patient education. Exercise intensity was based on baseline functional capacity and progressively adjusted throughout the program.

This approach is consistent with the ATS/ERS definition of pulmonary rehabilitation as a comprehensive intervention based on patient assessment, individualized exercise training, education, and behavior-change strategies, with the aim of improving physical and psychological condition and promoting long-term adherence to health-enhancing behaviors [32].

#### 2.2.2. Drug Therapy

All patients received daily Inhaled Corticosteroids [beclomethasone dipropionate: C_28_H_37_ClO_7_] 0.4 mg/mL in combination with two bronchodilators, such as one SABA [i.e., salbutamol: C_13_H_21_NO_3_] and one SAMA [anti subtype M3, i.e., ipratropium bromide: C_20_H_30_BrNO_3_] for 3 weeks. Pharmacological treatment was prescribed and adjusted according to the treating physician’s clinical judgment and the individual patient’s clinical condition. Oxygen therapy was administered when clinically indicated and according to individual oxygen requirements and medical prescription; in the event of an exacerbation or clinical deterioration, systemic corticosteroid therapy was administered according to clinical indication, including oral prednisone [C_21_H_26_O_5_] at 5 or 25 mg, or betamethasone [C_22_H_29_FO_5_] at 1.5 mg or with injectable methylprednisolone [C_22_H_30_O_5_] at 20 mg. Other supportive treatments and modifications of concomitant therapy were provided when clinically indicated according to standard clinical practice.

#### 2.2.3. Functional Response to Pulmonary Rehabilitation

Functional response was assessed using the Six-Minute Walk Test (6MWT)**,** performed before and after rehabilitation according to established American Thoracic Society recommendations [32]. Rehabilitation response was primarily assessed according to the change in 6 min walking distance (6MWD) from baseline to the end of the pulmonary rehabilitation program.

A change of 30 m or more was considered a clinically meaningful improvement. This threshold is consistent with the ERS/ATS technical standard on field walking tests, which identified a median minimal important difference (MID) of approximately 30 m across the available evidence [32]. The same threshold has also been applied in pulmonary rehabilitation studies; for example, Dajczman et al. [33] used an improvement of ≥30 m as the minimally important difference in patients undergoing pulmonary rehabilitation.

The three response categories used in the present analysis—non-responders (<20 m), moderate responders (20 to <120 m), and good responders (≥120 m)—were used to describe the magnitude of functional improvement and were not intended to represent alternative definitions of the MID.

#### 2.2.4. Lung Function and FEV1

FEV1 was considered primarily as a measure of respiratory impairment and disease severity, rather than as a specific predictor of the response to pulmonary rehabilitation. FEV1 data were available for 51 of the 102 patients in the primary cohort. This difference reflects the retrospective, real-world nature of the study. Spirometry was performed as part of routine clinical assessment and was not a study-specific measurement systematically repeated in all participants. Patients entered the rehabilitation program after hospitalization and clinical stabilization, and pulmonary function measurements therefore depended on the clinical assessments available in their medical records during the preceding or rehabilitation admission.

Consequently, complete pre- and post-rehabilitation FEV1 measurements were not available for the entire cohort. The 51 patients with available measurements were not selected according to their rehabilitation response or clinical outcome; rather, they represented those for whom sufficiently complete spirometric data were available in the clinical records. The available FEV1 measurements were therefore used primarily to characterize respiratory impairment and disease severity rather than to define rehabilitation response.

Mean FEV1 was 46.03 ± 22.78% predicted at admission and 60.15 ± 21.31% predicted at discharge. Although FEV1 increased during rehabilitation, the principal outcome of the present study was functional recovery, assessed by the change in 6MWD rather than by the change in FEV1.

This distinction reflects the multidimensional nature of COPD and pulmonary rehabilitation. COPD is increasingly recognized as a heterogeneous disease whose clinical expression extends beyond the degree of airflow limitation. Using data from the ECLIPSE cohort, Agustí et al. [34] highlighted substantial heterogeneity among patients with COPD and the contribution of multiple pulmonary and extrapulmonary factors. Agustí and Faner [2] subsequently proposed a broader paradigm in which COPD is considered beyond smoking and airflow obstruction, incorporating aging, environmental exposures, genetic susceptibility, inflammation, and other biological mechanisms.

The specific role of FEV1 in pulmonary rehabilitation was also addressed by Spruit and Franssen [31], who emphasized that FEV1 should not be used alone to identify rehabilitation responders and non-responders because clinically meaningful changes in exercise capacity, symptoms, and health status are not adequately captured by airflow limitation alone. Similarly, the official ATS/ERS statement by Spruit et al. [32] describes pulmonary rehabilitation as a comprehensive intervention targeting exercise capacity, symptoms, health status, and other patient-centered outcomes rather than lung function alone.

Our recent integrated model of precision pulmonary rehabilitation further supports this multidimensional interpretation. Russo et al. [35] proposed that responsiveness to pulmonary rehabilitation in COPD reflects the interaction of functional, biological, behavioral, immune, and environmental factors rather than a single clinical or biological determinant.

Accordingly, in the present study, FEV1 was used for respiratory and disease characterization, whereas change in 6MWD was selected as the principal measure of functional response to rehabilitation. FEV1 was therefore not interpreted as a specific predictor of rehabilitation response.

#### 2.2.5. Clinical Assessment

At admission, all participants underwent a comprehensive multidimensional assessment including demographic, clinical, respiratory, cognitive, psychological, functional, lifestyle, and immunological variables.

Clinical assessment included symptom burden, health status, and functional independence, evaluated using the Borg scale, Medical Research Council (MRC) dyspnea scale, Barthel Index, Activities of Daily Living (ADL), Instrumental Activities of Daily Living (IADL), Maugeri Foundation Respiratory Failure Questionnaire (MRF-26), St. George’s Respiratory Questionnaire (SGRQ), and Short Form-36 (SF-36).

Functional exercise capacity was assessed using the 6MWT, whereas cognitive performance was evaluated using the Mini-Mental State Examination (MMSE) and the Montreal Cognitive Assessment (MoCA).

Comorbidity burden and clinical complexity were assessed using the Cumulative Illness Rating Scale (CIRS) Severity and Comorbidity Indices, with particular attention to cardiovascular disease and type 2 diabetes mellitus.

Instrumental and physiological assessments included spirometry, including FEV1, pulse oximetry, electrocardiography, heart rate, blood pressure, arterial blood gas analysis (pO2, pCO2, and pH), SpO2, and SpCO2 measurements. Laboratory assessments included hematological and biochemical parameters, including electrolytes (Na+ and K+), hemoglobin, glucose, blood urea nitrogen, creatinine, bilirubin, and liver enzymes (ALT, AST, and γGT), as previously described [36].

#### 2.2.6. Psychological Assessment

Psychological status was evaluated using validated questionnaires.

Depressive symptoms were assessed using both the Beck Depression Inventory-II (BDI-II) and the Center for Epidemiologic Studies Depression Scale (CES-D). Anxiety symptoms were evaluated using the Zung Self-Rating Anxiety Scale (SAS).

#### 2.2.7. Coping Strategies

Behavioral adaptation to chronic disease was evaluated at admission before rehabilitation using the Brief COPE Inventory, a validated self-administered questionnaire developed by Carver [14] to assess cognitive and behavioral coping strategies adopted in response to stressful situations.

The present study focused on three coping dimensions considered particularly relevant for rehabilitation adherence: active coping, reflecting direct behavioral efforts aimed at solving or reducing the impact of stressful situations; planning, representing the tendency to develop concrete strategies and organize future actions to manage disease-related challenges; and self-distraction, describing temporary diversion of attention toward alternative activities in order to reduce emotional distress associated with chronic illness.

Each Brief COPE subscale consists of two items scored on a four-point Likert scale, yielding a maximum score of 8 points, with higher scores indicating greater use of the corresponding coping strategy.

Internal consistency of the selected subscales was evaluated using Cronbach’s alpha, with values greater than 0.72 indicating satisfactory reliability.

#### 2.2.8. Lifestyle Assessment

Lifestyle habits were assessed through standardized questionnaires.

Smoking exposure was evaluated by both self-reported smoking history and urinary cotinine, allowing participants to be classified as never smokers, former smokers, or current smokers as described previously [37].

Anthropometric assessment included measurement of body mass index (BMI), which was analyzed both as a continuous variable and according to obesity status (BMI ≥ 30 kg/m^2^).

Dietary habits focused on the habitual consumption of fresh fruit, vegetables, green leafy vegetables, according to a European Union (EU) validated questionnaire to 158 estimate food items intake [38]. No patient reported never consuming vegetables; data on vegetable consumption were missing for 25 patients.

#### 2.2.9. Immune Biomarkers Analysis

Peripheral venous blood samples were collected before rehabilitation.

Circulating lymphocyte subsets were quantified by flow cytometry including the following: CD3^+^ T lymphocytes, CD4^+^ T lymphocytes, CD8^+^ T lymphocytes, regulatory T cells and T helper subset, as described previously [36].

Torque Teno virus (TTV) plasma load was quantified by real-time quantitative PCR as previously described [26,36]. Based on previous studies, patients were classified according to a cut-off value of ≥4 log_10_ copies/mL, considered indicative of increased viral replication and reduced immune competence [26].

#### 2.2.10. Survival Analysis

Vital status was assessed after a 5-year follow-up. To this aim, follow-up was performed by tracing participants at their registered place of residence. Information regarding vital status and causes of death was obtained from the corresponding municipal records. Overall survival was calculated from the date of rehabilitation admission until death or the end of follow-up. Kaplan–Meier survival curves were generated according to rehabilitation response groups. To explore whether functional independence and comorbidity severity independently influenced long-term survival, stratified Cox proportional hazards regression models were performed including IADL and CIRS Severity Index as covariates while stratifying by rehabilitation response.

#### 2.2.11. Mediation Analysis

To investigate the role of coping mechanisms in the relationship between age at admission and treatment response, causal mediation analyses were conducted using the mediation package in R (version 4.4.2). Data were obtained from a sample of 74 patients. Age at admission was specified as the independent variable (X), whereas clinical outcome (Responder) was used as the dependent variable (Y), coded dichotomously (0 = no response; 1 = moderate or good response). Coping dimensions assessed with the Brief COPE questionnaire were examined individually as potential mediators (M). Specifically, the following subscales were evaluated: planning, active coping, acceptance, self-distraction, venting, use of instrumental support, use of emotional support, behavioral disengagement, and denial. Prior to analysis, each mediator was standardized using z-score transformation.

For each mediation model, two regression models were estimated. The mediator model consisted of a linear regression examining the association between age at admission and the coping variable (M~X). The outcome model consisted of a binary logistic regression evaluating the effects of age at admission and the mediator on clinical response status, coded as non-responder (0) versus moderate/good responder (1) (Y~X + M).

Mediation effects were estimated using the counterfactual framework implemented in the mediated function. The average causal mediation effect (ACME; indirect effect), average direct effect (ADE; direct effect), total effect, and proportion mediated were calculated. Ninety-five percent confidence intervals and *p*-values were obtained using nonparametric bootstrap percentile confidence intervals based on 1000 simulations. Statistical significance was defined as a two-sided *p* value < 0.05.

### 2.3. Secondary Study IMPROVE Pilot Trial

The IMPROVE (Psychological Intervention to Improve the COPD Patients’ Empowerment and Quality of Life) pilot study was a prospective randomized controlled trial conducted at IRCCS San Raffaele Roma and approved by the local Ethics Committee (Protocol No. 22/2017; ClinicalTrials.gov NCT03470025). It was conducted at a later stage of the research program in a separate cohort and was designed to investigate whether structured psychological support could modify coping-related characteristics and help maintain the benefits of pulmonary rehabilitation. The study enrolled 36 patients with COPD (GOLD I–II), an MMSE score ≥ 26, and a baseline six-minute walking distance (6MWD) ≥ 90 m. Participants were consecutively recruited and randomly allocated by block randomization to one of three groups: face-to-face plus telephone psychological intervention (F-TPI, n = 12), telephone psychological intervention (TPI, n = 12), or standard pulmonary rehabilitation alone (Control, n = 12). All participants underwent a 3-week multidisciplinary pulmonary rehabilitation program, with psychological support added according to the assigned intervention. The analytical role of IMPROVE differed from that of the primary observational study, which examined the association between coping strategies and functional response to pulmonary rehabilitation in patients with advanced COPD (GOLD III–IV). IMPROVE instead explored whether coping-related characteristics could be modified through structured psychological support and whether this approach could help sustain rehabilitation benefits over time. Its findings were therefore considered complementary and exploratory, providing preliminary interventional evidence on the potential modifiability of coping strategies. Given the small sample size and the different COPD severity of the participants, IMPROVE was not used to validate the associations observed in the primary cohort or to establish a causal relationship between coping strategies and functional improvement.

The psychological intervention combined psychoeducation with structured psychological and emotional support delivered over six months through face-to-face and/or telephone sessions. Participants underwent standardized pulmonary rehabilitation followed by multidimensional evaluation including pulmonary function, quality of life, psychological status, coping strategies, cognitive performance, and functional outcomes. Psychological assessment included the SCL-90-R, BDI-II, STAI-Y, Brief COPE, and MMSE. Detailed methodology has been reported previously [25].

### 2.4. Statistical Analysis

Values are reported as mean ± SD or median (Q1–Q3), as appropriate. Group comparisons were performed using one-way ANOVA or Kruskal–Wallis tests. Post hoc analyses were conducted using Bonferroni-corrected pairwise comparisons after ANOVA and Dunn–Bonferroni tests after Kruskal–Wallis analysis. Categorical variables were compared using Pearson’s χ^2^ test. Overall survival was evaluated using the Kaplan–Meier method, and differences between survival curves were assessed using the log-rank test. Multivariable survival analyses were performed using Cox proportional hazards regression. To investigate whether coping strategies mediated the association between age at admission and treatment response, causal mediation analyses were performed using the mediation package. Age at admission was specified as the independent variable, treatment response (non-responder vs. moderate/good responder) as the binary outcome, and each Brief COPE subscale was evaluated separately as a potential mediator. Mediation effects were estimated using the counterfactual framework with nonparametric bootstrap percentile confidence intervals based on 1000 simulations. The average causal mediation effect (ACME), average direct effect (ADE), total effect, and proportion mediated were reported. Missing data were handled using complete-case analysis. Consequently, the number of participants included varied across analyses according to data availability for the variables of interest. No imputation procedures were applied. All statistical analyses were performed using R version 4.4.2. Two-sided *p* values < 0.05 were considered statistically significant.

## 3. Results

### 3.1. Baseline Characteristics

#### Study Population

A total of 102 elderly patients with advanced COPD completed the multidisciplinary baseline assessment before pulmonary rehabilitation. The cohort included 56 women (54.9%) and 46 men (45.1%), with a mean age of 72.6 ± 8.7 years. All patients had severe airflow limitation (GOLD stage III–IV) and underwent a standardized pulmonary rehabilitation program.

Functional response was evaluated according to the change in six-minute walking distance (Δ6MWD) as good responders (Δ6MWD ≥ 120 m; n = 40), moderate responders (20–119 m; n = 29), and non-responders (<20 m; n = 33).

### 3.2. Clinical Characteristics

Significant baseline differences were observed across response groups for several demographic and clinical variables (Table 1).

Good responders were significantly younger than non-responders (67.99 ± 8.16 vs. 74.85 ± 8.02 years, *p* = 0.012), while no difference was found between moderate responders and the other groups. Sex distribution did not differ among rehabilitation response groups, with women representing approximately half of the participants in each group (50.0%, 58.3%, and 56.7% in non-, moderate, and good responders, respectively; *p* = 0.844).

Body mass index did not differ significantly among groups.

Functional status at admission was strongly associated with rehabilitation outcome. Both Barthel Index and ADL and IADL scores were markedly lower in non-responders than in moderate and good responders (all *p* < 0.01), indicating a substantially greater degree of baseline disability among patients who subsequently failed to achieve clinically meaningful functional improvement.

Disease burden also differed between groups. Non-responders reported significantly higher dyspnea severity at admission, with Borg scores approaching the upper end of the scale 9.0 (9.0–9.0), compared with both moderate 7.0 (7.0–8.0) and good responders 7.0 (7.0–9.0) (*p* < 0.001). Likewise, respiratory disability assessed by the MRF-26 questionnaire was significantly worse in non-responders than in moderate responders (*p* < 0.001), whereas St. George’s Respiratory Questionnaire scores showed only a non-significant trend toward poorer health status in non-responders.

Comorbidity severity, assessed by the CIRS Severity Index, was also higher among non-responders than good responders (*p* = 0.036), while the overall burden of comorbid diseases (CIRS Comorbidity Index) did not differ significantly between groups.

### 3.3. Psychological and Cognitive Characteristics

No significant differences were observed among rehabilitation response groups in cognitive performance, depressive symptoms, anxiety, or health-related quality of life (Table 1). MMSE and MoCA scores, as well as CES-D, Zung SAS, and SF-36 scores, were comparable across groups.

### 3.4. Oxidative Stress and Inflammatory Biomarkers

Baseline biomarkers of oxidative stress and systemic inflammation were largely comparable across rehabilitation response groups (Table 1).

DNA damage measured by comet assay (Tail Intensity %) tended to be higher in good responders than in non-responders; however, the difference did not reach statistical significance (*p* = 0.299). Similarly, circulating concentrations of malondialdehyde (MDA), 8-hydroxy-2′-deoxyguanosine (8-OHdG), and interleukin-6 (IL-6) did not differ significantly among groups.

### 3.5. Lifestyle Characteristics According to Pulmonary Rehabilitation Response

Baseline lifestyle characteristics were broadly comparable across pulmonary rehabilitation response groups (Table 2). Smoking status, assessed by urinary cotinine, showed a similar distribution of never, current, and former smokers across the three rehabilitation response categories (*p* = 0.960). Regular vegetable consumption tended to be more frequent among moderate (40.0%) and good responders (35.7%) than among non-responders (7.1%), although the difference did not reach statistical significance (*p* = 0.091). Fresh fruit consumption was also similar across groups (*p* = 0.487). All participants reported no consumption of spirits, while wine intake was uniformly low, not exceeding approximately one glass per day (two half-glasses). Likewise, all patients described a sedentary lifestyle, reporting no more than two hours of physical activity per week, consisting almost exclusively of short walks. Overall, lifestyle variables showed limited variability within the cohort and were not significantly associated with pulmonary rehabilitation responsiveness.

Data are presented as number and percentage. Smoking status was assessed by urinary cotinine. Regular vegetable consumption was available for 62 participants (14 non-responders, 20 moderate responders, and 28 good responders). Fresh fruit consumption was available for 63 participants (15 non-responders, 20 moderate responders, and 28 good responders). Categorical variables were compared using Pearson’s χ^2^ test.

### 3.6. Immune Biomarkers

Baseline immune profiling did not reveal statistically significant differences among non-responders, moderate responders, and good responders (Table 3). Absolute circulating.

CD3+, CD4+, and CD8+ lymphocyte counts tended to increase progressively across rehabilitation response categories, with the highest values consistently observed in good responders, although these trends did not reach statistical significance.

Similarly, no significant differences were found for regulatory T cells (CD25+ CD127−), Th1 and Th2 subsets, CD4/CD8 ratio, or α7 nicotinic acetylcholine receptor expression. However, a progressive reduction in Th17 cells was observed from non-responders to good responders (38.68 ± 22.20, 24.49 ± 22.78, and 13.59 ± 14.25, respectively; *p* = 0.093). Although not statistically significant, this progressive reduction was accompanied by a gradual increase in the Treg/Th17 ratio, suggesting a shift toward a more balanced immune regulatory profile among patients achieving the greatest functional improvement.

Torque Teno virus (TTV) viremia, considered a surrogate marker of global immune competence rather than a direct respiratory pathogen, was comparable among rehabilitation response groups. Mean plasma TTV load (≥4 log_10_ copies/mL) showed no significant differences between non-responders, moderate responders, and good responders (*p* = 0.341), indicating that baseline systemic immune competence, as assessed by circulating TTV levels, was not independently associated with functional response to pulmonary rehabilitation.

The prevalence of elevated Torque Teno virus (TTV) viremia (≥4 log_10_ copies/mL), a surrogate marker of immune competence, did not differ significantly among rehabilitation response groups (*p* = 0.136).

Similarly, the distribution of peripheral T-cell subsets was comparable among rehabilitation response groups. No significant differences were observed in the percentages of CD3+, CD4+, CD8+, or regulatory T lymphocytes (all *p* > 0.05). Although non-responders tended to present lower CD3+ T-cell percentages and higher regulatory T-cell levels than responders, these trends did not reach statistical significance.

### 3.7. Behavioral Adaptation and Coping Strategies and Pulmonary Rehabilitation Response

Marked differences in coping strategies emerged across pulmonary rehabilitation response groups (Table 4). Patients achieving the greatest functional improvement reported a behavioral profile characterized by greater engagement in active coping strategies. In particular, active coping (*p* = 0.019), acceptance (*p* = 0.002), and especially planning (*p* < 0.001) progressively increased from non-responders to good responders, with Bonferroni analysis showing significantly higher scores in good responders than in non-responders. A similar trend was observed for positive reframing, although it did not reach statistical significance (*p* = 0.079).

Several coping strategies related to social support also differed between groups. Emotional support was significantly higher in both moderate and good responders than in non-responders (*p* = 0.004), while instrumental support was higher in moderate responders than in non-responders (*p* = 0.011). Self-distraction also increased with rehabilitation responsiveness (*p* = 0.001), with both moderate and good responders scoring significantly higher than non-responders. Likewise, venting was more frequently adopted by good responders than by non-responders (*p* = 0.008).

Interestingly, behavioral disengagement was highest among good responders (*p* = 0.001), who scored significantly higher than both non-responders and moderate responders. Denial was also more frequent in moderate responders than in non-responders (*p* = 0.028), although no difference was observed between moderate and good responders.

In contrast, religion, humor, substance use, self-blame, and positive reframing did not differ significantly across rehabilitation response groups.

Sex-stratified analyses revealed distinct coping patterns associated with rehabilitation response (Table 5). Among women, responders reported significantly higher scores for self-distraction (5.70 ± 1.64 vs. 3.82 ± 1.47, *p* = 0.002), active coping (6.80 ± 1.56 vs. 5.36 ± 1.57, *p* = 0.013), and planning (6.67 ± 1.56 vs. 5.36 ± 2.11, *p* = 0.038) compared with non-responders. No significant differences were observed for the remaining coping domains, although positive reframing, religion, instrumental support, denial, and self-blame showed trends toward higher scores among responders.

In men, the association between coping style and rehabilitation response was more pronounced. Responders scored significantly higher than non-responders for venting (5.14 ± 1.39 vs. 3.64 ± 0.81, *p* = 0.002), active coping (7.05 ± 1.50 vs. 5.73 ± 1.95, *p* = 0.039), behavioral disengagement (3.91 ± 1.85 vs. 2.64 ± 0.81, *p* = 0.038), emotional support (4.64 ± 1.18 vs. 3.36 ± 0.92, *p* = 0.004), acceptance (4.95 ± 1.50 vs. 3.73 ± 1.10, *p* = 0.022), and planning (6.86 ± 1.81 vs. 4.91 ± 1.64, *p* = 0.005). No significant differences were found for the remaining Brief COPE domains.

### 3.8. Mediation Analysis of Coping Strategies

To investigate whether coping strategies explained the association between age and pulmonary rehabilitation responsiveness, mediation analyses were performed for the nine Brief-COPE dimensions (Table 6).

Among all coping strategies, planning was the only significant mediator of the relationship between age and rehabilitation response. The average causal mediation effect (ACME) was statistically significant (estimate = −2.04 × 10^−5^, 95% CI −1.52 × 10^−3^ to −1.71 × 10^−10^, *p* = 0.042), whereas the average direct effect (ADE) showed only borderline significance (*p* = 0.060). The total effect of age remained significant (estimate = −7.71 × 10^−5^, 95% CI −1.68 × 10^−3^ to −2.62 × 10^−8^, *p* = 0.010). Planning was the only coping dimension showing evidence compatible with mediation of the association between age and rehabilitation response; however, given the observational design, multiple mediation analyses, and wide confidence interval of the mediated proportion, this finding should be considered exploratory (95% CI 0.9–99.3%, *p* = 0.044).

No significant mediation effects were observed for the remaining coping dimensions, including active coping, acceptance, self-distraction, venting, use of instrumental support, use of emotional support, behavioral disengagement, and denial. For all these strategies, ACME estimates were not statistically significant (*p* > 0.05), and the confidence intervals for the proportion mediated included zero, indicating no evidence of indirect effects.

Conversely, significant direct effects of age on rehabilitation responsiveness were consistently observed across most models, including those including active coping (*p* = 0.030), acceptance (*p* = 0.018), self-distraction (*p* = 0.032), venting (*p* = 0.014), use of instrumental support (*p* = 0.008), use of emotional support (*p* = 0.006), behavioral disengagement (*p* = 0.020), and denial (*p* = 0.014). Across all models, the total effect of age remained statistically significant (*p* = 0.006–0.024), supporting age as an independent determinant of pulmonary rehabilitation responsiveness.

### 3.9. Five-Year Survival

To evaluate the long-term prognostic relevance of pulmonary rehabilitation responsiveness, all participants were prospectively followed for five years after completion of the rehabilitation program. Kaplan–Meier survival curves stratified according to rehabilitation response are presented in Figure 1.

Kaplan–Meier analysis demonstrated a significant association between rehabilitation response and long-term survival (log-rank Mantel–Cox χ^2^ = 6.57, df = 2, *p* = 0.038). Patients classified as good responders consistently showed the highest probability of survival throughout the five-year follow-up, whereas non-responders exhibited the poorest survival. Moderate responders displayed an intermediate survival pattern, lying between the other two groups (Figure 1).

Patients classified as good responders consistently exhibited the highest probability of survival throughout the follow-up period, whereas non-responders showed the lowest survival estimates, with moderate responders displaying an intermediate pattern. Although this gradient suggests a clinically meaningful relationship between functional improvement after rehabilitation and long-term prognosis, the survival curves partially overlapped and the overall differences did not reach statistical significance.

To investigate whether baseline multidimensional clinical characteristics independently influenced mortality beyond rehabilitation responsiveness, stratified Cox proportional hazards regression analyses were performed using rehabilitation response group as the stratification factor (Table 7).

Neither functional independence, assessed by the IADL scale, nor comorbidity burden, evaluated using the CIRS Severity Index, emerged as independent predictors of five-year all-cause mortality. In the multivariable model, IADL showed a hazard ratio (HR) of 1.11 (95% CI: 0.91–1.35, *p* = 0.325), whereas the CIRS Severity Index showed an HR of 0.70 (95% CI: 0.07–7.37, *p* = 0.770). Similar findings were obtained in the univariable analyses (IADL: HR = 1.04, 95% CI: 0.89–1.21, *p* = 0.630; CIRS Severity Index: HR = 0.29, 95% CI: 0.06–1.51, *p* = 0.142). (Table 6).

### 3.10. IMPROVE Pilot Randomized Controlled Trial

Baseline demographic, clinical, and psychological characteristics were comparable among the three randomized groups, confirming successful allocation and the absence of significant pre-intervention differences (Table 8 and Supplementary Table S1).

However, differences emerged during follow-up. At six months, both psychological intervention groups maintained significantly greater rehabilitation benefits than controls, with better respiratory health-related quality of life on the MRF-26 (TPI vs. CTRL, *p* = 0.003; F-TPI vs. CTRL, *p* < 0.0001) (Table 7; Supplementary Table S3). Psychological support was also associated with reduced depressive symptoms, state and trait anxiety, and phobic anxiety, together with improvements in the SF-36 Physical Role domain and Q-LES-Q scores. Behavioral adaptation improved, with increased active coping, emotional support, and instrumental support and reduced denial and self-blame, particularly in the TPI group. In contrast, controls showed a decline in positive reframing and humor (Table 8; Supplementary Table S4). These findings suggest that adaptive coping can be modified through psychological intervention and may contribute to maintaining the benefits of pulmonary rehabilitation.

## 4. Discussion

The present study suggests that behavioral adaptation, assessed through coping strategies, is associated with pulmonary rehabilitation responsiveness in elderly patients with advanced COPD. Patients with greater functional improvement reported higher levels of active coping and planning and, to a lesser extent, self-distraction. In contrast, pulmonary function, smoking status, nutritional and lifestyle variables, immune biomarkers, and most psychological measures showed limited differences between response groups.

A relevant finding was obtained from the mediation analysis. Planning was the only coping dimension showing evidence compatible with mediation of the association between age and rehabilitation response; however, given the observational design, multiple mediation analyses, and wide confidence interval of the mediated proportion, this finding should be considered exploratory. The other coping strategies did not show significant mediation effects. This finding suggests that differences in the ability to anticipate and organize responses to rehabilitation demands may partly contribute to the lower response observed with increasing age, although the mediation analysis should be considered exploratory given the sample size.

Behavioral adaptation and pulmonary rehabilitation

Pulmonary rehabilitation improves exercise tolerance, dyspnea, health status and quality of life in COPD, although individual responses remain heterogeneous [4,5,6,7,39]. Our findings indicate that this variability may also reflect behavioral characteristics.

Patients with greater functional improvement reported higher active coping and planning. Both strategies involve problem solving, goal setting and active engagement with treatment and have been associated with self-management, self-efficacy and adherence in chronic diseases [14,15,16]. Planning may be particularly relevant in COPD because it involves anticipating problems and organizing appropriate responses.

The association with planning was further supported by the mediation analysis. Its significant mediation of the age–response relationship suggests that planning ability may be one of the behavioral factors contributing to differences in rehabilitation response with age. This interpretation is consistent with evidence from COPD self-management programs. Individualized action plans combined with brief education can improve recognition of symptom deterioration and promote timely treatment, while reducing COPD-related emergency department visits and hospital admissions [40]. Although action plans are not equivalent to the planning construct measured by the Brief COPE, both involve anticipating problems and translating intentions into concrete actions.

These findings should also be considered within the broader psychological context of COPD. Moretta et al. [41] described a bidirectional relationship between respiratory disease and psychological factors, in which anxiety and depression may influence symptom perception, physical activity, treatment adherence and disease management, while the physical limitations and burden of COPD may in turn contribute to psychological distress. This interaction is particularly relevant in rehabilitation, where motivation, engagement and the ability to cope with persistent dyspnea and functional limitations may influence how patients participate in and benefit from treatment. Moretta et al. also emphasized the value of integrating psychological assessment and intervention within multidisciplinary pulmonary rehabilitation rather than considering psychological factors as secondary to respiratory impairment [41].

Recent evidence provides further support for this interpretation. Vitacca et al. [42], in a multicenter cohort of 975 patients undergoing inpatient pulmonary rehabilitation, found that psychiatric and neurocognitive comorbidities were common and were associated with differences in rehabilitation outcomes. Although pulmonary rehabilitation improved symptoms, dyspnea, functional independence and exercise capacity across groups, patients with cognitive impairment showed the poorest multidimensional response, while a formal psychiatric diagnosis was independently associated with a lower probability of achieving the predefined composite response. These findings reinforce the view that psychological and cognitive characteristics may contribute to heterogeneity in rehabilitation outcomes and support the relevance of including these dimensions in the assessment of rehabilitation potential [42].

Our findings extend this perspective by focusing not on psychiatric diagnoses or cognitive impairment, but on specific coping processes. In particular, the association of planning and active coping with greater functional improvement suggests that behavioral characteristics may provide additional information even among patients with advanced COPD undergoing the same rehabilitation program. This distinction is important, because coping strategies should not be considered equivalent to psychiatric comorbidity; rather, they may represent behavioral processes through which patients engage with rehabilitation demands.

Self-distraction was also associated with rehabilitation response. Although generally considered an avoidant coping strategy [43], limited use may help patients temporarily shift attention away from persistent symptoms without necessarily interfering with participation in rehabilitation. This finding should nevertheless be interpreted cautiously.

Sex-specific analyses showed that planning and active coping were associated with response in both women and men, while some additional associations differed by sex. Women showed an association with self-distraction, whereas men showed associations with acceptance, venting, emotional support and behavioral disengagement. These differences are broadly consistent with previous studies reporting more frequent use of emotion-focused coping among women and problem-focused or instrumental strategies among men [19,20]. The possible relationship between emotion-focused coping and psychological distress has also been described [21,44]. These findings should be regarded as exploratory, particularly because of the limited sample size.

The sex-related findings may also be viewed in light of recent rehabilitation data. Vitacca et al. [42] reported a higher prevalence of psychiatric disorders among women admitted to pulmonary rehabilitation, particularly depressive disorders. Although their study examined psychiatric and neurocognitive comorbidities rather than coping strategies, the findings support the possibility that psychological factors and sex may interact in shaping rehabilitation trajectories. The present results therefore add a behavioral perspective to this literature, while the different study designs and constructs examined do not allow a direct comparison.

Overall, the results are compatible with the concept of behavioral adaptation, defined as the capacity to maintain adaptive behavior despite persistent physical and psychological stress [22,23]. In this context, coping strategies may provide information on how patients engage with the demands of rehabilitation rather than representing fixed psychological traits. The Brief COPE provides an assessment of specific coping responses that may contribute to adaptive functioning, but it does not directly measure resilience as a broader psychological construct. Therefore, the observed associations should be interpreted in terms of specific coping processes rather than as a direct assessment of resilience.

Pulmonary function, lifestyle and immune biomarkers

Most conventional clinical and biological variables showed limited ability to distinguish rehabilitation response. Pulmonary function was not significantly associated with outcome, despite slightly higher FEV_1_ values among good responders. This observation is consistent with previous evidence showing that baseline lung function alone has limited value in identifying patients who respond to pulmonary rehabilitation. Augustin et al. [45] found that comprehensive lung function assessment did not allow rehabilitation responders and non-responders to be reliably distinguished, while Troosters et al. [46] similarly showed that baseline physiological characteristics alone were insufficient to explain differences in response to exercise training. More recently, Spruit and Franssen [31] emphasized that baseline FEV_1_ should not be used to identify responders or non-responders to pulmonary rehabilitation, as clinically relevant improvements in exercise capacity, symptoms and health status may occur across different degrees of airflow limitation. Age, sex, BMI, smoking status and dietary habits were also broadly comparable across groups. These findings are consistent with the multidimensional nature of COPD, in which airflow limitation does not fully capture differences in symptoms, exercise capacity, comorbidities and other clinical characteristics, as highlighted by Agustí et al. [34] and Agustí and Faner [2]. In line with this broader perspective, Russo et al. [35] proposed that rehabilitation responsiveness reflects the interaction of functional, biological, behavioral, immune and environmental factors rather than a single clinical or biological determinant.

Immune biomarkers showed a similar pattern. Neither TTV viremia nor circulating T-cell subsets differed according to rehabilitation response. Although high TTV load has been associated with immune exhaustion, genomic instability and mortality in elderly patients with severe COPD, these findings suggest that TTV may be more informative as a marker of immune competence and long-term prognosis than as a determinant of short-term functional recovery. Vitiello et al. [36] recently showed that high TTV viremia was associated with T-cell depletion, increased regulatory T cells, altered α7nAChR expression and shorter five-year survival in the same clinical population, whereas Treg frequency, rather than TTV load itself, emerged as the independent predictor of mortality. Similarly, de Iure et al. [38] reported associations between smoking, TTV burden, immune alterations, oxidative DNA damage and long-term outcomes, further supporting the interpretation of TTV as part of a broader biological vulnerability profile rather than as a specific marker of rehabilitation response. Russo et al. [35], integrating these findings within a broader biological model of pulmonary rehabilitation, emphasized that rehabilitation responsiveness cannot be explained by a single immune or biological marker but rather reflects the interaction of functional, biological, behavioral and environmental factors. Taken together, these observations may explain why immune biomarkers associated with disease progression and biological aging do not necessarily distinguish patients according to their functional response during a short rehabilitation program.

### 4.1. Functional Reserve and Long-Term Outcomes

Functional independence and comorbidity burden were associated with rehabilitation response, suggesting that preserved functional reserve may facilitate participation in a demanding multidisciplinary program.

Patients classified as good responders also had better five-year survival, with significant differences among the three response groups (log-rank *p* = 0.019). However, after adjustment, baseline functional independence and comorbidity burden did not independently predict mortality. Thus, the survival analysis should be interpreted as showing an association between rehabilitation response and long-term outcome rather than a direct prognostic effect of the baseline variables examined.

### 4.2. From Observational Findings to Intervention

The observational results provided the rationale for the IMPROVE pilot randomized trial. In that study, adding structured psychological support to pulmonary rehabilitation was associated with a better maintenance of rehabilitation benefits over six months, together with improvements in psychological well-being, quality of life and several coping dimensions. The changes included increased active coping, emotional support and instrumental support and reduced denial and self-blame.

These results are consistent with the observational findings but do not establish that changes in coping directly caused better clinical outcomes. The small sample and pilot design also limit the strength of the conclusions. Nevertheless, the findings indicate that coping behaviors can be influenced through structured psychological and educational support and may be relevant to the maintenance of rehabilitation benefits.

### 4.3. Strengths and Limitations

A strength of the study is the multidimensional assessment of rehabilitation response, bringing together clinical, functional, psychological, lifestyle, immunological and virological variables within the same cohort. This approach allowed us to examine coping strategies in the context of the broader clinical and biological characteristics of patients undergoing pulmonary rehabilitation, rather than considering them in isolation. The later IMPROVE pilot study provided complementary interventional evidence that psychological support can influence coping-related characteristics and help maintain some of the benefits achieved with rehabilitation. However, because IMPROVE involved a separate and smaller population, it should be considered supportive rather than a validation of the present observational findings.

Several limitations should also be acknowledged. The observational study was conducted at a single rehabilitation center and included elderly patients with advanced COPD, which may limit the generalizability of the findings to other populations and rehabilitation settings. Some clinical, biological and lifestyle variables were available only for subsets of patients, reducing the power of individual analyses. The mediation analysis was performed in a relatively small subgroup and should therefore be interpreted as exploratory. In addition, the observational design does not allow causal conclusions about the relationship between coping, age and rehabilitation response, and residual confounding cannot be excluded. The number of events was also limited for analyses involving longer-term outcomes. Finally, behavioral adaptation was assessed through specific Brief-COPE dimensions rather than with a dedicated measure of resilience. The findings therefore concern coping processes and should not be interpreted as a direct measure of resilience. Future studies should confirm these observations in larger, preferably prospective, cohorts and consider more comprehensive assessment of coping, resilience, self-efficacy and cognitive or executive functions.

### 4.4. Clinical Implications

The findings suggest that psychological and behavioral characteristics may add useful information to the conventional assessment of patients entering pulmonary rehabilitation. In particular, planning and active coping were associated with greater functional improvement and may help clinicians recognize patients who could need additional support in organizing and managing rehabilitation-related demands. This does not mean that Brief-COPE should be used as a clinical prediction tool, nor should psychological assessment replace the usual evaluation of respiratory impairment, functional status, dyspnea and comorbidities. Rather, a brief assessment of coping may help identify patients who could benefit from additional psychological, educational or self-management support. This interpretation is consistent with the growing recognition that psychological and cognitive factors are relevant components of pulmonary rehabilitation and may influence how patients engage with and benefit from treatment. Moretta et al. [41] discussed the clinical significance of psychiatric disorders in COPD, while Vitacca et al. [42] reported that psychiatric and neurocognitive comorbidities are common among patients undergoing inpatient pulmonary rehabilitation and are associated with differences in rehabilitation outcomes. The present findings concern a related but distinct aspect: specific coping strategies rather than psychiatric diagnoses or cognitive impairment. These constructs should not be conflated. Any assessment of coping should complement, rather than replace, standard clinical and functional evaluation. The IMPROVE pilot provides a rationale for further testing structured psychological support, but larger controlled studies are needed to establish whether interventions targeting coping strategies improve functional outcomes.

## 5. Conclusions

Pulmonary rehabilitation response in elderly patients with advanced COPD is unlikely to be explained by respiratory impairment alone. In this cohort, specific coping strategies, particularly active coping and planning, were associated with greater functional improvement. Planning was also the only Brief-COPE dimension showing a significant indirect effect in the relationship between age and rehabilitation response. Given the sample size and observational design, this finding should be regarded as exploratory and requires confirmation in larger prospective studies.

Conventional clinical, lifestyle and immune variables showed limited differences between response groups, supporting a multidimensional view of rehabilitation responsiveness. The separate IMPROVE pilot study provides preliminary interventional support for the possibility that coping-related characteristics can be influenced by structured psychological support and that such support may help maintain rehabilitation benefits. However, IMPROVE was not designed to validate the observational findings, and it remains uncertain whether changes in coping directly translate into greater functional recovery or longer-term benefits. Future studies should therefore investigate whether integrating behavioral and psychological assessment into pulmonary rehabilitation can improve patient selection, individualization of support and maintenance of rehabilitation outcomes.

## 6. Patents

No patents resulted from the work reported in this manuscript.

## Figures and Tables

**Figure 1:**
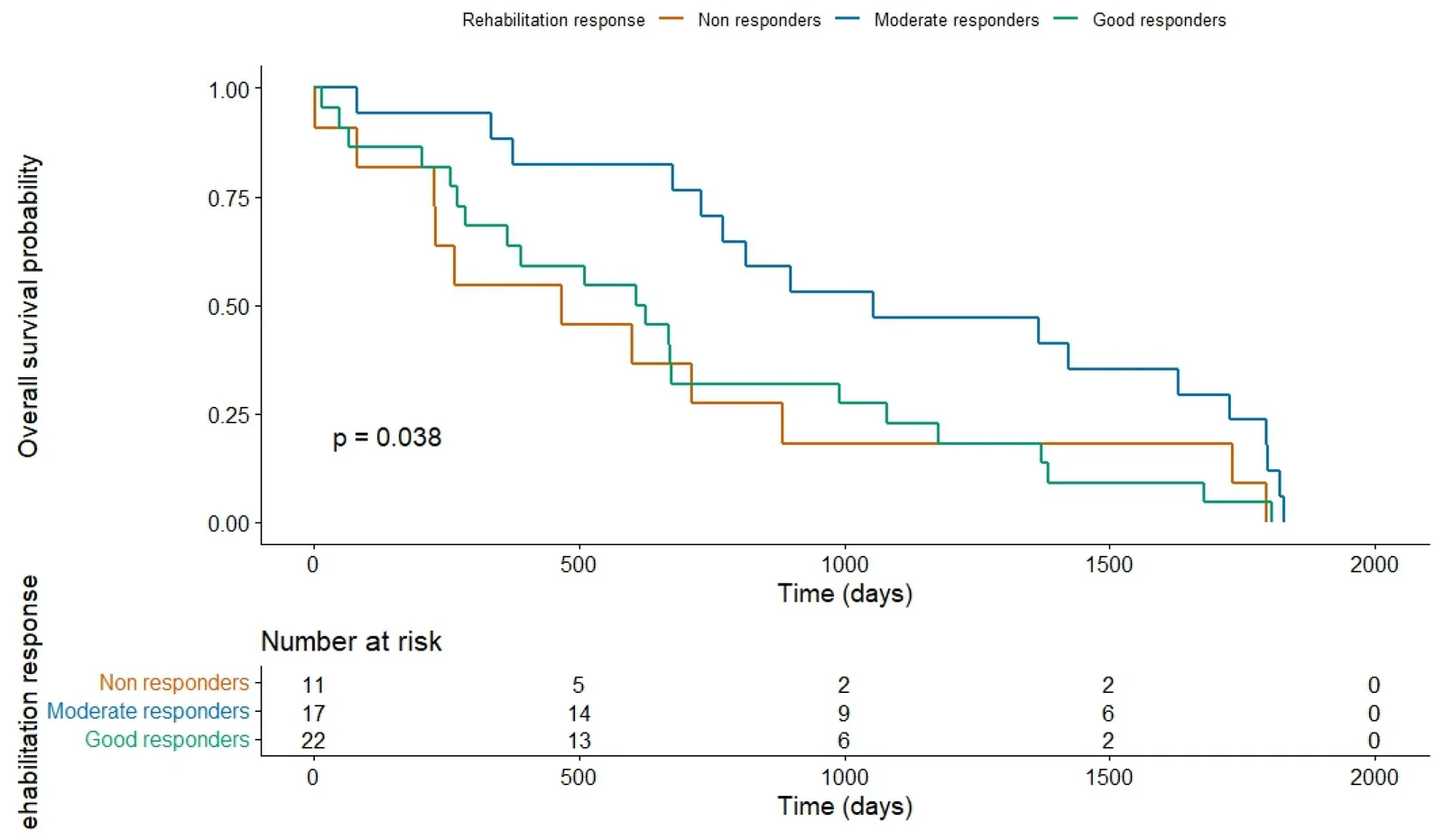
Kaplan–Meier survival curves according to rehabilitation response groups. Figure has been improved using ChatGPT. (ChatGPT plus (GPT-5.5)).

**Table 1:** Baseline characteristics according to pulmonary rehabilitation response.

Variable	Non Responders	Moderate Responders	Good Responders	*p*- Value *	Post Hoc **
Age (years)	74.85 ± 8.02	72.57 ± 8.15	67.99 ± 8.16	0.012	NR vs. GR (*p* = 0.014);
BMI (kg/m^2^)	24.2 (21.0–26.0)	24.1 (20.7–30.5)	27.2 (24.2–34.1)	0.061	NS
Barthel Index	42.0 (27.0–59.0)	83.0 (77.0–85.0)	82.0 (65.5–87.2)	<0.001	NR vs. MR (*p* < 0.001); NR vs. GR (*p* < 0.001);
Borg Dyspnea Score	9.0 (9.0–9.0)	7.0 (7.0–8.0)	7.0 (7.0–9.0)	<0.001	NR vs. MR (*p* < 0.001); NR vs. GR (*p* < 0.001)
MRF-26	84.6 (76.9–84.6)	71.2 (65.4–76.9)	76.9 (69.2–80.8)	<0.001	NR vs. MR (*p* < 0.001)
St George’s Score	57.5 (48.2–64.8)	44.0 (34.0–57.0)	47.0 (33.0–58.0)	0.028	NR vs. GR (*p* = 0.049);
ADL	2.00 (2.00–5.25)	6.00 (6.00–6.00)	6.00 (6.00–6.00)	<0.001	NR vs. MR (*p* < 0.001); NR vs. GR (*p* < 0.001)
IADL	5.0 (3.0–11.0)	13.0 (11.0–15.0)	13.0 (11.0–15.0)	0.008	NR vs. MR (*p* = 0.021); NR vs. GR (*p* = 0.009)
CIRS Severity	1.72 ± 0.26	1.60 ± 0.21	1.53 ± 0.22	0.036	NR vs. GR (*p* = 0.030)
CIRS Comorbidity	3.0 (2.0–4.0)	2.0 (2.0–4.0)	3.00 (2.0–4.0)	0.513	NS
MMSE	27.0 (25.0–28.8)	28.0 (27.0–30.0)	28.0 (25.75–30.0)	0.148	NS
MoCA	27.4 (24.0–28.9)	29.0 (26.0–30.0)	26.2 (24.25–28.0)	0.076	NS
CES-D	10.0 (5.50–12.0)	7.0 (0.0–14.5)	7.00 (1.0–17.0)	0.447	NS
Zung	26.0 (24.0–29.0)	25.0 (22.0–31.5)	25.0 (23.0–34.0)	0.690	NS
SF-36 General Health	70.0 (62.5–77.5)	80.0 (65.0–85.0)	75.0 (67.5–80.0)	0.328	NS
SF-36 Mental Health	64.0 (56.0–68.0)	64.0 (60.0–69.0)	64.0 (56.0–68.0)	0.954	NS
Tail Intensity (%)	17.66 ± 4.59	18.41 ± 8.52	21.02 ± 8.22	0.299	NS
MDA	1.56 (39.0–46.8)	37.2 (30.8–44.2)	37.37 (32.3–42.4)	0.278	NS
8-OHdG	26.57 ± 12.01	24.20 ± 12.78	25.64 ± 11.79	0.882	NS
IL-6 (pg/mL)	52.5 (13.1–05.6)	33.4 (0.00–45.6)	23.6 (0.00–75.4)	0.179	NS

Abbreviations: NR, non-responders; MR, moderate responders; GR, good responders; SD, standard deviation; NS, not significant. Mean ± SD or median (Q1–Q3), as appropriate; * ANOVA one way—Kruskal–Wallis test; ** Bonferroni-corrected pairwise comparisons after ANOVA and Dunn-Bonferroni tests after Kruskal–Wallis analysis.

**Table 2:** Lifestyle characteristics according to pulmonary rehabilitation response.

Variable	Non-Responders (n = 33)	Moderate Responders (n = 29)	Good Responders (n = 40)	*p*-Value
Smoking status (cotinine), n (%)				
Current smokers	11 (33.3)	12 (41.4)	15 (37.5)	0.960
Former smokers	17 (51.5)	13 (44.8)	18 (45.0)	
Never smokers	5 (15.2)	4 (13.8)	7 (17.5)	
Regular vegetable consumption, n (%) *Yes*	1/14 (7.1)	8/20 (40.0)	10/28 (35.7)	0.091
Fresh fruit consumption, n (%) *Yes*	10/15 (66.7)	16/20 (80.0)	23/28 (82.1)	0.487

Values are n (%) or n/N (%), as indicated.

**Table 3:** Baseline immune biomarkers according to pulmonary rehabilitation response.

Variable	Non-Responders	Moderate Responders	Good Responders	*p*-Value *	Post Hoc **
CD3+ T cells (n/mm^3^)	604.0 (466.5–850.3)	950.0 (761.5–1168.5)	1034.5 (618.3–1425.5)	0.074	NS
CD3+ CD4+ T cells (n/mm^3^)	438.5 (353.3–555.0)	567.0 (338.5–628.5)	676.0 (422.5–979.5)	0.129	NS
CD3+ CD8+ T cells (n/mm^3^)	04.5 (82.75–170.5)	228.0 (147.5–459.0)	277.5 (138.3–391.3)	0.082	NS
Treg	0.50 (0.00–1.45)	0.30 (0.20–0.70)	0.40 (0.08–0.62)	0.847	NS
Th1 (%)	3.00 (0.70–7.00)	1.45 (0.75–2.15)	2.25 (1.70–5.60)	0.441	NS
Th2 (%)	3.7 (3.00–16.45)	11.75 (1.33–17.3)	5.25 (0.55–13.18)	0.608	NS
Th17 (%)	38.68 ± 22.20	24.49 ± 22.78	13.59 ± 14.25	0.093	NS
α7-nAChR+ CD4+ cells (%)	10.0 (2.50–61.5)	87.00 (10.00–96.0)	71.0 (33.0–93.0)	0.126	NS
α7-nAChR+ CD4+ cells (MFI)	1839.0 (772.0–3073.5)	5673.0 (1657.5–6186.0)	2617.0 (931.0–6074.0)	0.247	NS
TTV load (>4 log_10_ copies/mL)	1.00 (1.00–1.00)	1.00 (0.00–1.00)	1.00 (0.00–1.00)	0.136	NS
CD4/CD8 ratio	3.10 (2.46–4.42)	2.46 (1.62–3.23)	2.95 (2.02–5.97)	0.434	NS
Treg/Th17 ratio	0.01 (0.00–0.06)	0.01 (0.01–0.02)	0.02 (0.01–0.17)	0.726	NS

Abbreviations: SD, standard deviation; Treg, regulatory T cells; Th1, T helper 1; Th2, T helper 2; Th17, T helper 17; a7-nAChR, alpha 7 nicotinic receptors; MFI, mean fluorescence intensity; TTV, Torque Teno virus; NS, not significant. mean ± SD or median (Q1–Q3), as appropriate; * ANOVA one way—Kruskal Wallis test; ** Bonferroni-corrected pairwise comparisons after ANOVA and Dunn-Bonferroni tests after Kruskal–Wallis analysis.

**Table 4:** Brief COPE scores according to pulmonary rehabilitation response.

Variable	Non Responders (NR) (n = 20)	Moderate Responders (MR) (n = 24)	Good Responders (GR) (n = 30)	*p* Value	Variable
Positive reframing	4.10 ± 1.37	4.96 ± 1.49	5.17 ± 1.93	0.079	NS
Self-distraction	3.85 ± 1.23	5.13 ± 1.42	5.57 ± 1.85	0.001	NR < MR (*p* = 0.027); NR < GR (*p* = 0.001)
Venting	3.70 ± 0.86	4.58 ± 1.28	4.90 ± 1.54	0.008	NR < GR (*p* = 0.006)
Instrumental support	3.90 ± 0.79	5.08 ± 1.59	4.63 ± 1.22	0.011	NR < MR (*p* = 0.008)
Active coping	5.65 ± 1.73	6.58 ± 1.89	7.00 ± 1.31	0.019	NR < GR (*p* = 0.016)
Denial	3.05 ± 1.36	4.17 ± 1.49	4.00 ± 1.46	0.028	NR < MR (*p* = 0.039)
Religion	3.90 ± 1.48	4.79 ± 1.84	4.70 ± 1.56	0.150	NS
Humor	3.50 ± 1.05	4.04 ± 1.52	3.60 ± 1.30	0.332	NS
Behavioral disengagement	2.75 ± 1.07	2.75 ± 1.39	4.20 ± 1.79	0.001	GR > NR (*p* = 0.004); GR > MR (*p* = 0.002)
Emotional support	3.60 ± 0.88	4.83 ± 1.46	4.53 ± 1.17	0.004	NR < MR (*p* = 0.004); NR < GR (*p* = 0.028)
Substance use	2.30 ± 0.92	2.17 ± 0.48	2.47 ± 1.01	0.433	NS
Acceptance	4.20 ± 1.06	4.71 ± 1.57	5.67 ± 1.56	0.002	NR < GR (*p* = 0.002)
Planning	5.05 ± 1.93	6.25 ± 1.85	7.10 ± 1.35	<0.001	NR < GR (*p* < 0.001)
Self-blame	3.85 ± 1.46	4.25 ± 1.48	4.70 ± 1.53	0.146	NS

Values are presented as mean ± SD. Higher scores indicate greater use of the corresponding coping strategy (maximum score = 8). Overall group comparisons were per-formed using one-way ANOVA. Cronbach’s α was >0.72 for all three subscales.

**Table 5:** Brief-COPE strategies showing significant sex-specific differences in pulmonary rehabilitation response.

Brief-COPE Strategy	Females (F)	Males (M)	*p* Value
Self-distraction	✓	—	F: 0.002
Active coping	✓	✓	F: 0.013; M: 0.039
Venting	—	✓	M: 0.002
Emotional support	—	✓	M: 0.004
Acceptance	—	✓	M: 0.022
Planning	✓	✓	F: 0.038; M: 0.005
Behavioral disengagement	—	✓	M: 0.038

*✓ indicates a statistically significant association between the Brief-COPE strategy and pulmonary rehabilitation response in the respective sex; — indicates that no statistically significant association was observed. F, females; M, males. p-values are reported for each sex separately*.

**Table 6:** Causal mediation analysis evaluating the role of Brief-COPE dimensions in the association between age and pulmonary rehabilitation responsiveness (n = 74).

Coping Strategy	ACME (Average), Estimate (95% CI)	*p*-Value	ADE (Average), Estimate (95% CI)	*p*-Value	Total Effect Estimate (95% CI)	*p*-Value	Proportion Mediated (%)	*p*-Value
Planning	**−2.04 × 10^−5^ (−1.52 × 10^−3^ to −1.71 × 10^−10^)**	0.042	**−5.67 × 10^−5^ (−7.61 × 10^−4^ to 1.23 × 10^−4^)**	**0.060**	**−7.71 × 10^−5^ (−1.68 × 10^−3^ to −2.62 × 10^−8^)**	0.010	26.5	0.044
Active coping	**−6.83 × 10^−6^ (−8.72 × 10^−4^ to 1.64 × 10^−5^)**	**0.212**	**−4.22 × 10^−5^ (−1.18 × 10^−3^ to −9.68 × 10^−10^)**	0.030	**−4.90 × 10^−5^ (−1.60 × 10^−3^ to −1.18 × 10^−8^)**	0.010	**13.9**	**0.218**
Acceptance	**−1.09 × 10^−5^ (−7.66 × 10^−4^ to 5.13 × 10^−5^)**	**0.184**	**−4.99 × 10^−5^ (−1.28 × 10^−3^ to −2.34 × 10^−8^)**	0.018	**−6.08 × 10^−5^ (−1.61 × 10^−3^ to −4.36 × 10^−8^)**	0.006	**17.9**	**0.190**
Self-distraction	**−8.93 × 10^−6^ (−1.10 × 10^−3^ to 7.77 × 10^−6^)**	**0.086**	**−3.37 × 10^−5^ (−9.62 × 10^−4^ to −1.19 × 10^−9^)**	0.032	**−4.26 × 10^−5^ (−1.41 × 10^−3^ to −8.54 × 10^−9^)**	0.012	**20.9**	**0.098**
Venting	**−3.47 × 10^−6^ (−2.75 × 10^−4^ to 1.57 × 10^−3^)**	**0.794**	**−7.06 × 10^−5^ (−2.74 × 10^−3^ to −9.09 × 10^−9^)**	0.014	**−7.41 × 10^−5^ (−1.61 × 10^−3^ to −2.83 × 10^−9^)**	0.024	**4.7**	**NA**
Use of instrumental support	**−1.68 × 10^−6^ (−1.40 × 10^−4^ to 8.10 × 10^−4^)**	**0.832**	**−7.59 × 10^−5^ (−2.14 × 10^−3^ to −3.87 × 10^−8^)**	0.008	**−7.76 × 10^−5^ (−1.67 × 10^−3^ to −3.65 × 10^−8^)**	0.012	**2.2**	**0.820**
Use of emotional support	**9.05 × 10^−6^ (−5.07 × 10^−5^ to 1.19 × 10^−3^)**	**0.446**	**−8.90 × 10^−5^ (−2.68 × 10^−3^ to −1.35 × 10^−8^)**	0.006	**−7.99 × 10^−5^ (−1.70 × 10^−3^ to −1.15 × 10^−8^)**	0.012	**−11.3**	**0.458**
Behavioral disengagement	**−5.01 × 10^−6^ (−2.88 × 10^−4^ to 4.20 × 10^−4^)**	**0.710**	**−7.87 × 10^−5^ (−1.70 × 10^−3^ to −1.16 × 10^−8^)**	0.020	**−8.37 × 10^−5^ (−1.38 × 10^−3^ to −1.16 × 10^−8^)**	0.020	**6.0**	**0.698**
Denial	**−2.11 × 10^−6^ (−1.64 × 10^−4^ to 4.87 × 10^−4^)**	**0.688**	**−5.35 × 10^−5^ (−2.00 × 10^−3^ to −2.75 × 10^−8^)**	0.014	**−5.56 × 10^−5^ (−1.60 × 10^−3^ to −2.39 × 10^−8^)**	0.016	**3.8**	**0.684**

Abbreviations: ACME, average causal mediation effect; ADE, average direct effect; CI, confidence interval.

**Table 7:** Stratified Cox proportional hazards regression analysis evaluating the independent association of baseline functional independence (IADL) and comorbidity burden (CIRS Severity Index) with five-year all-cause mortality. Rehabilitation response group was included as the stratification factor. Hazard ratios (HRs) with 95% confidence intervals (CIs) are reported. The multivariable model included baseline IADL and CIRS Severity Index as independent variables. Rehabilitation response group was included as a stratification factor and therefore no hazard ratio was estimated for this variable.

Variable	Hazard Ratio (HR)	95% Confidence Interval	*p* Value
**Multivariable model**			
IADL	1.11	0.91–1.35	0.325
CIRS Severity Index	0.70	0.07–7.37	0.770
**Univariable models**			
IADL	1.04	0.89–1.21	0.630
CIRS Severity Index	0.29	0.06–1.51	0.142

**Table 8:** Quantitative evidence from the IMPROVE pilot study supporting the present investigation.

Outcome	Main Result	Effect Size/Values	*p* Value
**Baseline comparability**	No significant differences among the randomized groups.	Comparable demographic, clinical and psychological characteristics	NS
**Pulmonary rehabilitation response**	All participants responded to the 4-week pulmonary rehabilitation program before randomization.	Clinically meaningful improvement in exercise capacity and respiratory status	Within-group *p* < 0.005
**Maintenance of PR benefits (MRF-26)**	Control patients deteriorated, whereas both intervention groups maintained or further improved respiratory health-related quality of life.	A2: CTRL = +10.0 ± 13.6 TPI = −5.44 ± 3.46 F-TPI = −12.39 ± 10.67	TPI vs. CTRL = 0.003 F-TPI vs. CTRL < 0.0001
**SF-36**	Role limitations due to physical health markedly improved after psychological intervention.	TPI ≈ 5.2-fold improvement F-TPI ≈ 4.0-fold improvement vs. CTRL	Significant
**Depression (BDI-II)**	Reduction in depressive symptoms in both intervention groups; worsening trend in controls.	Improvement in both intervention groups	TPI vs. CTRL = 0.014 F-TPI vs. CTRL = 0.009
**Anxiety (STAI-Y)**	Reduction in state and trait anxiety.	Improvement in both intervention groups	*p* = 0.005
**Phobic anxiety (SCL-90-R)**	Reduced psychological distress.	Decrease after intervention	*p* = 0.040
**Coping strategies (Brief COPE)**	Increased adaptive coping.	↑ Instrumental support ↓ Denial Greater empowerment	All *p* < 0.05
**Exercise tolerance (6MWT)**	Psychological intervention prolonged rehabilitation benefits.	Intervention groups: +15% Control: −15%	

Abbreviations. PR, pulmonary rehabilitation; CTRL, control group; TPI, telephone psychological intervention; F-TPI, face-to-face plus telephone psychological intervention; MRF-26, Maugeri Respiratory Failure Questionnaire; BDI-II, Beck Depression Inventory-II; STAI-Y, State–Trait Anxiety Inventory; SCL-90-R, Symptom Checklist-90-Revised; 6MWT, 6 min walk test. *↑ increase, ↓ decrease*.

## Data Availability

Data are available in Zenodo at https://zenodo.org/records/22809159 Accessed on 17 September 2026.

## Supplementary Materials

The following supporting information can be downloaded at: https://www.mdpi.com/article/10.3390/arm94050074/s1, Table S1. Demographic and clinical characteristics of the 36 subjects at baseline, during the Pulmonary Rehabilitation program, stratified by the three treatment groups (Control Group: CTRL; Telephone Group: TPI; Face-Telephone Group: F-TPI). Table S2. MRF-26 data of study subjects (N = 31), after pulmonary rehabilitation (PR) and after 6 months of IMPROVE study, stratified by the three treatment groups. We reported Δ: Δ1= value at discharge - baseline value at hospitalization; Δ2 = value after 6 months - value at discharge. Table S3. Psychological parameters: Assessment for Anxiety and Depression, of the subjects who completed the study (N = 31), stratified by the three treatment groups. Table S4. Assessment of Coping strategies: Brief-COPE values in subjects, before rehabilitation and 6 months after discharge, stratified for the three treatment groups.

## Abbreviations

The following abbreviations are used in this manuscript:

6MWT	6-Minute walking Test
ADL	Activities of Daily Living
BDI-II	Beck Depression Inventory-II
BMI	Body Mass Index
CD	Cluster of Differentiation
CES-D	Center for Epidemiologic Studies Depression Scale
CIRS	Cumulative Illness Rating Scale
COPD	Chronic Obstructive Pulmonary Disease
CRP	C reactive protein
DMSO	Dimethylsulfoxide
FEV1	Forced Expiratory Volume in 1 s
GOLD	Global initiative for chronic Obstructive Lung Diseases
IADL	Instrumental Activities of Daily Living
IL-6	interleukine-6
MMSE	Mini-Mental State Examination
MRC	modified Medical Research Council (Dyspnoea Scale)
MRF-26	Maugeri Respiratory Failure 26 questions
PLT	Platelets
PR	Pulmonary Rehabilitation
QoL	Quality of Life
RBC	Red Blood Cells
SF-36	36-Item Short Form Health Survey—General Health domain;
TTV	Torque Teno Virus
VZV	Varicella Zoster Virus
WBC	white blood cells
ZUNG	Zung Self-Rating Depression Scale
