# Supplementary material for: Behavioral Adaptation and Response to Pulmonary Rehabilitation in Older Patients with Advanced COPD: Beyond Clinical and Immune Biomarkers
Source: Adv Respir Med. 2026 Oct 19;94(5):74. doi: 10.3390/arm94050074 (PMC13701965; doi:10.3390/arm94050074)
Supplement: Supplementary file 1 [file arm-94-00074-s001.zip › arm-4602836-supplementary.pdf]

Table S1. Demographic and clinical characteristics of the 36 subjects at baseline, during the Pulmonary Rehabilitation program, stratified by the three treatment groups (Control Group: CTRL; Telephone Group: TPI; Face-Telephone Group: F-TPI).

Demographic and Epidemiological variables	**CTRL (N=12)**	**TPI (N=12)**	**F-TPI (N=12)**	***p***
**Gender**				
Males	7 (58.33%)	3 (25.0%)	6 (50%)	*ns*
Females	5 (41.67%)	9 (75.0%)	6 (50%)	
**Age (years)**	73.17± 8.43	73.83±5.17	67.92±8.27	*ns*
**Smoking habit**				
Non-smoker	2 (16.67%)	2 (16.67%)	3 (25.0%)	*ns*
Current smoker	3 (25.0%)	2 (16.67%)	6 (50.0%)	
former smoker	7 (58.33%)	8 (66.67%)	3 (25.0%)	
**Body Mass Index**	26.95±5.93	28.02±7.00	25.77±7.43	*ns*
**Obesity (new BMI≥30)**	4 (33.33%)	3 (25.0%)	2 (16.67%)	*ns*
**MMSE**	27.83±1.64	27.67±1.44	27.50±1.51	*ns*
**BDI-II**	12.8±7.37	16.70±9.33	18.26±12.20	*ns*
**6MWD (meters)**	135.8±83.61	133.3±72.53	151.7±79.98	*ns*
**MRF-26**	52.24±18.95	56.73±14.02	56.39±22.82	*ns*

Table S2. MRF-26 data of study subjects (N = 31), after pulmonary rehabilitation (PR) and after 6 months of IMPROVE study, stratified by the three treatment groups. We reported Δ: Δ1= value at discharge - baseline value at hospitalization; Δ2 = value after 6 months - value at discharge.

	**CTRL (N=10)**	**TPI (N=12)**	**F-TPI (N=9)**	***Anova p values ¥***	
				TPI vs CTRL	*F-TPI vs CTRL*
**MRF-26**					
After PR	34.92±17.77	37.82±20.21	32.30±24.54		
After 6 months	43.46±16.32	32.37±20.25	20.94±21.77		
Δ1	-17.32±17.02**	-18.91±25.17*	-23.08±27.20*	*ns*	*ns*
Δ2	10.0±13.60*	-5.44±3.46***	-12.39± 10.67**	0.003	*p<0.0001*

*Paired *t* Test: statistically significant values of *Δ* are highlighted in bold (p<0.005)

¥ Univariate anova with Bonferroni correction

Table S3. Psychological parameters: Assessment for Anxiety and Depression, of the subjects who completed the study (N = 31), stratified by the three treatment groups.

	CTRL (N=10)	TPI (N=12)	F-TPI (N=9)	*Anova p values ¥*	
				TPI vs CTRL	*F-TPI vs CTRL*
**BDI-II**					
Before	12.83±6.73	16.70±9.33	21.23±12.20		
After 6 months	16.80±10.96	12.67±6.035	16.11±10.06		
Δ	4.0±5.35	-4.03±5.61	-5.12±7.34	0.014	0.009
**STAI-Y-Y-2 (Trait)**					
Before	41.20±12.97	43.17±10.50	42.22±13.84		
After 6 months	45.20±13.59	36.83±6.81	40.22±13.38		
Δ	4.0±4.88	-6.33±9.72	-2.0±3.81	0.005	0.05
**STAI-Y-Y-1 (State)**					
Before	41.10±14.80	42.58±10.59	40.0±14.65		
After 6 months	46.20±15.48	36.33±7.41	36.56±13.16		
Δ	5.10±7.11	-6.25±6.59	-3.87±9.67	0.005	0.05
***Phobic anxiety* -SCL-90**					
Before	0.23±0.20	0.52±0.48	0.48±0.65		
After 6 months	0.33±0.37	0.30±0.39	0.32±0.53		
Δ	0.10±0.27	-0.22±0.25	-0.16±0.15	0.04	0.04

**Paired T Test: the statistically significant Δ values are highlighted in bold (p <0.005)*

¥ ANOVA test with Bonferroni correction

Table S4. Assessment of Coping strategies: Brief-COPE values in subjects, before rehabilitation and 6 months after discharge, stratified for the three treatment groups.

	**CTRL (N=10)**		**TPI (N=12)**		**F-TPI (N=9)**		***Anova p values ¥***	
							TPI vs CTRL	*TPI vs CTRL*
	**Before**	**6 Months**	**Before**	**6 Months**	**Before**	**6 Months**		
Positive Reframing	6.20±2.15	5.80±2.30*	5.58±1.83	6.00±1.76	5.56±2.30	6.11±2.09		
Self-distraction	5.40±2.06	*5.20*±2.66	*5.42*±1.88	*5.67*±1.44	4.89±2.37	5.44±1.94		
Venting	3.90±1.29	*4.0*±1.56	5.50±1.73	4.0±1.81	3.33±1.58	3.44±1.42		
Use of instrumental support	4.30±2.11	*4.0*±2.26	4.17±1.75	5.58±1.14*	5.00±1.22	6.11±0.33*	*0.017*	*0.05*
Active coping	5.80±1.81	*5.70*±2.11	5.25±1.76	6.25±1.66*	6.67±1.80	6.79±0.98		
Denial	4.10±1.97	*5.0*±2.45	4.25±1.96	3.17±1.47**	2.78±0.98	2.33±0.71	*0.004*	*0.05*
Religion	3.70±2.00	*3.90*±2.18	5.42±2.43	4.92±2.35	3.79±2.49	4.0±2.65		
Humor	5.10±1.99	3.80±1.87**	4.92±2.33	5.25±2.38	4.0±1.87	4.22±1.39		
Behavioral disengagement	3.90±1.59	3.40±1.65	4.25±1.77	3.83±1.19	2.56±0.73	3.44±1.59		
Use of emotional support	4.20±1.92	4.60±2.12	3.75±1.14	5.08±1.08***	4.56±1.42	6.00±1.41*		

Substance use	2.20±0.63	2.0±0.0	2.08±0.29	2.0±00	2.0±0.0	2.22±0.67		
Acceptance	6.0±1.70	6.20±1.62	6.17±2.17	6.25±1.91	5.78±1.92	5.33±1.87		
Planning	6.80±1.32	6.40±1.71	5.67±2.10	6.58±1.38	6.67±1.73	6.33±1.58		
Self-blame	4.40±1.51	4.40±1.96	4.92±2.50	4.0±1.91*	5.33±1.80	3.11±1.45**	*n.s*	*0.004*

Paired T Test: the statistically significant Δ values are highlighted in bold ($p < 0.005$)

¥ Anova uni varied with Bonferroni correction
